# Visual Information Alone Changes Behavior and Physiology during Social Interactions in a Cichlid Fish (*Astatotilapia burtoni*)

**DOI:** 10.1371/journal.pone.0020313

**Published:** 2011-05-25

**Authors:** Chun-Chun Chen, Russell D. Fernald

**Affiliations:** 1 Neurosciences Program, Stanford University, Stanford, California, United States of America; 2 Department of Biology Sciences, Stanford University, Stanford, California, United States of America; Biodiversity Insitute of Ontario - University of Guelph, Canada

## Abstract

Social behavior can influence physiological systems dramatically yet the sensory cues responsible are not well understood. Behavior of male African cichlid fish, *Astatotilapia burtoni,* in their natural habitat suggests that visual cues from conspecifics contribute significantly to regulation of social behavior. Using a novel paradigm, we asked whether visual cues alone from a larger conspecific male could influence behavior, reproductive physiology and the physiological stress response of a smaller male. Here we show that just seeing a larger, threatening male through a clear barrier can suppress dominant behavior of a smaller male for up to 7 days. Smaller dominant males being “attacked” visually by larger dominant males through a clear barrier also showed physiological changes for up to 3 days, including up-regulation of reproductive- and stress-related gene expression levels and lowered plasma 11-ketotestesterone concentrations as compared to control animals. The smaller males modified their appearance to match that of non-dominant males when exposed to a larger male but they maintained a physiological phenotype similar to that of a dominant male. After 7 days, reproductive- and stress- related gene expression, circulating hormone levels, and gonad size in the smaller males showed no difference from the control group suggesting that the smaller male habituated to the visual intruder. However, the smaller male continued to display subordinate behaviors and assumed the appearance of a subordinate male for a full week despite his dominant male physiology. These data suggest that seeing a larger male alone can regulate the behavior of a smaller male but that ongoing reproductive inhibition depends on additional sensory cues. Perhaps, while experiencing visual social stressors, the smaller male uses an opportunistic strategy, acting like a subordinate male while maintaining the physiology of a dominant male.

## Introduction

During social interactions, individuals receive multiple forms of sensory information and use these signals to establish and maintain dominance hierarchies [1], [2], [3]. In many species, individuals change their physiological responses during social interactions. For example, in the bluegill sunfish, *Lepomis macrochirus*, the presence of a mature male can inhibit the sexual maturation of juvenile males [4]. It is also known that visual signals during social interactions can evoke changes in behavior patterns [5], [6], circulating hormone concentration [7], [8], [9], monoaminergic activity [10], [11], and neuropeptide gene expression [12]. Many fish species appear to rely on visual signals during social encounters particularly to maintain their social hierarchy [2], [7], [13], [14], [15]. However, the importance of visual information in influencing social status, relative to other senses, is unclear.

Teleost fish, and in particular an African cichlid, *Astatotilapia burtoni*, live in an environment well suited for visual signaling [16] and have an excellent visual system, with high resolution trichromatic vision [17]. In *A. burtoni*, a fraction (10–30%) of males form a dynamic social hierarchy centered around resource guarding and hence are called territorial males [16]. When territorial males are in physical proximity to each other, they fight more or less continuously over territory ownership and boundaries. During such male-male interaction, visual information appears to play an important role in regulation of the dominance hierarchy. Territorial males, have brightly colored bodies, and perform numerous agonistic and reproductive behaviors [16], [18]. They are reproductively competent with large, spermiated gonads and have a constellation of physiological markers of dominance in the brain-pituitary-gonad axis [19]. The reproductive dominance of territorial males includes higher gonadotropin releasing hormone (GnRH1) levels in the brain [20] higher GnRH type 1 receptor levels in the pituitary [21], and higher circulating androgen levels [22]. In contrast, losers of territorial fights, called non-territorial males, school with females, are drably colored and are reproductively suppressed. Non-territorial males, similar to socially subordinate animals of other species, also have elevated cortisol levels in response to social stress of territorial male behavior [23]. In fish, the hypothalamus-pituitary-interrenal (HPI) axis regulates the response to stress via control of cortisol production [24], [25]. Non-territorial males have lower level of corticotropin-releasing factor (CRF) and CRF type 1 receptor (CRF-R1) in the brain, and higher expression of corticotropin-releasing factor binding protein (CRFBP) in the pituitary [26].

The behavioral and physiological characteristics related to social status *in A. burtoni* offer a unique opportunity to assess how visual signals alone could influence the behavior and physiology of social status. Although visual interactions have been studied in other species, including fish, many of these used stationary “dominant animal” models, or presented aggressive behavior via a video display [5]. We devised a novel paradigm that simulates an intrusion by one male into another male's territory. This allowed testing the role of active visual signals alone on the behavior, appearance, hormone concentrations and gene expression levels in *A. burtoni*, so we could isolate and identify the role of visual cues on the brain-pituitary-gonad and the HPI axis. We measured both short and long term effects on expression levels of the GnRH and CRF family of ligand encoding genes, key receptor genes and associated binding proteins.

We found that upon discovering that a larger (4X) dominant male apparently occupied the same territorial shelter, the smaller dominant male changed both his behavior and physiology. Over a one-week period, social behavior and chromatic body patterns were significantly reduced in response to viewing the larger animal. However, these visual signals alone did not mimic the full effect that occurs when animals interact physically. Interestingly, the smaller experimental subject reduced outward signs of his previous dominant state (e.g. color, behavior), but the concomitant physiological markers in both the reproductive and stress axis were not changed after a full week. These data suggest that seeing a large male can regulate the behavior of smaller males, but that full reproductive inhibition depends on additional sensory cues. While experiencing social stressors visually, the subject acts as an opportunist, sustaining subordinate behavior and thereby reducing or avoiding threats from the larger conspecific. However, the visual threats do not completely suppress the subject because he retains his own dominant reproductive physiology profile. In sum, visual signals alone from a social suppressor initiate a descent in social status, triggering subordinate behavior, but do not produce the full suite of physiological changes typically caused by social suppression.

## Materials and Methods

### Ethics statement

All work was performed in compliance with the animal care and use guidelines of the Stanford University Administrative Panel on Laboratory Animal Care. This study has the approval of the Stanford Administrative Panel on Laboratory Animal Care (Protocol 9882).

### Animals

We used an African cichlid fish species, *Astatotilapia burtoni*, originally derived from a wild-caught population, raised in aquaria under conditions matched to their native equatorial habitat in Lake Tanganyika, Africa (pH 8, 28°C) and fed once a day with cichlid pellets and flakes (AquaDine, Healdsburg, CA). Fish were kept in a 12-hr light, 12-hr dark cycle including 10 minutes of transitional twilight each morning and evening. Aquaria had a gravel substrate and terracotta pots were placed in each aquarium to facilitate establishment of territories. Fish used in this study were sexually mature females and territorial males. Prior to experimentation, animals were kept in a community tank with 2–3 territorial males, 4–6 non-territorial males and 7–10 females. Males were tagged with unique colored bead combinations to allow individuals to be identified during behavioral observations. To be classified as a territorial male for experiments, the subject must have shown a dominance index (DI  =  [number of aggressive behaviors + reproductive behaviors- fleeing]/minute [20]) greater than 2 for at least two weeks. DI for each individual was calculated daily for two weeks. Behavioral observations took place during 10-minute observation periods within 1 to 1.5 hours after light onset.

Subject fish were, on average, 6.56±0.045 cm long (n = 60; mean ± standard error (SE)) and weighed an average of 7.87±0.16 g (mean ± SE). Animals were randomly assigned to experimental (N = 10) and control (N = 10) subjects in each experimental group. There were no significant differences between the control and experimental subjects in length (one-way analysis of variance, F_(1, 58)_ = 0.164, *p* = 0.687) or weight (F_(1, 58)_ = 0.599, *p* = 0.442). To maximize the effect of social suppression, we chose the stimulus fish (average 29.22±1.2 g and 9.91±0.1 cm long) to be approximately four-times larger in size than the subject, a choice based on extensive preliminary experiments (data not shown). Size differences were significant between all subjects and the stimuli fish in their initial weights (F_(1, 88)_ = 2031.491, *p*<0.001) and lengths (F_(1, 88)_ = 1852.321, *p*<0.001).

### Behavioral paradigm

**Figure 1:**
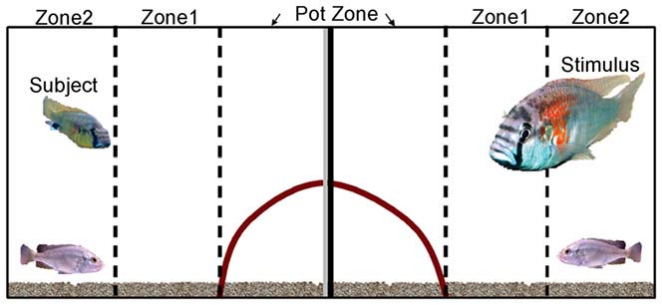
Sketch showing the aquarium used for the behavioral paradigm. An experimental tank (45l.) was divided in half with a watertight, clear divider (gray mid-line) and a removable opaque barrier (black mid-line). The small male fish in the left compartment is the subject and the large male fish (∼ 4 times larger) in the right compartment is the stimulus. A half terra cotta pot was cut in half and placed so that both the stimulus and subject “shared” the same shelter (dark curve). Note that this “shared” shelter was hemisected by both center dividers. A layer of gravel covered the bottom of the tank and the dotted lines identify three zones in each compartment used to record animal position.

The goal of the experimental design was to allow the large and small fish initially to inhabit a shared space with each one remaining dominant. To achieve this, the subject and stimulus fish were placed on opposite sides of a sealed, clear barrier that split the 45 liter tank in half. The sealed barrier prevented water flow and transmission of olfactory signals between the two chambers. Adjacent to the clear barrier was a removable opaque barrier. The tank was constructed to provide each animal a “shared” shelter comprised of a half 10 cm diameter terracotta flower pot. Usually, the animals would occupy the shelter under the half pot as their home territory where they built a nest for courting and spawning with females. However, this half pot was bisected longitudinally so that ¼ of the pot was on each side of the barrier. Thus, the shelter was halved with barriers between the 2 sections (see Figure 1). This design allowed both the clear and opaque barriers to hemi-sect the shelter. With the opaque barrier in place, the two dominant males each occupied a half shelter, and, importantly, neither animal was aware of nor could interact with the animal on the other side of the opaque barrier. This preserved normal dominant male behavior in subject and stimulus fish. One appropriately sized female was placed with each male. The subject and stimulus fish were habituated in this new testing environment for two days, during which time each behaved as a normal territorial male would by digging the substrate from their shelter, courting the female in their half of the tank and performing typical courtship and territorial male behaviors. After habituation, the opaque barrier between the compartments was removed and the sealed clear barrier remained in place during the remaining experimental period. Behavioral observations of both stimulus and subject were performed within 1 to 1.5 hours after light onset each subsequent morning. During the observations, the experimental subject fish and stimulus fish could see one-another but could not have any physical or olfactory or other contact across the sealed clear divider. Control experiments consisted of all the same conditions, except that no large male was in the adjoining half tank.

Behavioral acts were counted during 10-minute observation periods immediately before, immediately after, 1 hour after, and at every 24 hours after removing the opaque barrier in three separate test groups for 1 day, 3 days or 7 days. Behaviors were also recorded and scored each day until sacrifice for these conditions. Behavioral observations included tabulating aggressive, submissive and reproductive behavior, as well as the “shelter entry” frequency and the time spent close to the pot, as their home territory. The location relative to the pot of the fish was tabulated as being in one of the three zones as shown in Figure 1.

The data collected included the dominance index (DI), calculated from reproductive, aggressive and subordinate behaviors as described above. Reproductive behaviors tabulated included courting, spawning and digging (e.g., nesting) behaviors. The aggressive behaviors measured include threat displays, chasing, and border defense behaviors. Subordinate behaviors including fleeing from threatened attacks were also recorded, as were changes in body coloration and eye bar expression during interactions [16]. After behavioral observations were completed, the total body weight, length and gonad size were recorded.

### Circulating hormone levels

As noted, there were three experiments lasting 1, 3 and 7 days respectively. At the end of each experiment, subject and control animals were sacrificed. Immediately before sacrifice, blood samples (from 50 to 100 µl) were collected from the caudal vein of each male using a heparinized needle following well established laboratory procedures: Blood samples were obtained within 3 min of capture to ensure that any acute stress associated with drawing blood did not influence the measured cortisol levels [23]. Plasma was separated by centrifugation and stored at –80°C until assayed. The concentrations of cortisol, testosterone and 11-ketotestosterone (11-KT) in the plasma were measured using an enzyme-linked immuno-sorbent assay (ELISA; cortisol correlate-EIA kit and testosterone correlate-EIA kit Assay Designs, Inc., Ann Arbor, MI, USA, and 11-KT EIA kit, Cayman Chemical, Ann Arbor, MI, USA). We followed the protocol provided by the manufacturer for normalization and transformed measurements of the circulating hormone by the natural logarithmic function.

### Abundance of stress-related and reproduction-related genes in the brain

To understand the molecular consequences of visual encounters, we measured mRNA expression levels of several genes related to social status changes in the *A. burtoni* brain and pituitary gland using real time polymerase chain reactions (RT-PCR). After rapid decapitation, brains and pituitary glands were taken from males and immediately put into lysis buffer (RNeasy Micro Kit, Qiagen Inc., Valencia, CA), homogenized and stored at −80°C. Total RNA was extracted from samples following a standard protocol (RNeasy Micro Kit, Qiagen Inc., Valencia, CA). 1.0 μg total RNA was reverse transcribed (SuperScript II RNase H reverse transcriptase; Invitrogen, Carlsbad, CA) to cDNA in each sample. RT-PCR was performed to measure mRNA abundance using primers specific for *A. burtoni* target gene mRNAs (Table 1), which were designed using Primer3 (http://frodo.wi.mit.edu/primer3/) and Vector NTI (Invitrogen, CA). The Gene expression measured in this study included CRF (Genbank accession number: EF363131), CRFBP (GQ433718), two types of CRF receptors (type1 (CRF-R1: GQ433716) and type 2 (CRF-R2: GQ433717), somatostatin (AY585720), arginine vasotocin (AVT: AF517935), AVT receptor (AF517936), three types of gonadotropin-releasing hormone (GnRH1 (HBU31865), GnRH2 (L27435) and GnRH3 (S63657), and two types of GnRH receptors (type1 receptor (AY705931) and type 2 receptor (AY028476). The relative amounts of actin (JF826504), previously cloned from *A. burtoni*, did not significantly differ among the experimental and control groups. Thus, acitn is an appropriate housekeeping gene in this study and could be used to control for sample differences in total cDNA content. Polymerase chain reactions were performed (iCycler; Bio-Rad, Hercules, CA), and reaction progress in 30 μl reaction volumes was monitored by fluorescence detection at 490 nm during each annealing step. Reactions contained 2x IQ SYBR® Green SuperMix (Bio-Rad), 10 µM of each primer, and 1 ng cDNA (RNA equivalent). Reaction conditions were 1 min at 95°C; then 40 cycles of 30 s at 95°C, 30 s at 60°C, and 30 s at 72°C; followed by a melting curve analysis over the temperature range from 95°C to 4°C. All samples were run in duplicate.

**Table 1:** PCR Primers for *A. burtoni* target genes used in this study

Gene	GenBanck Access No.	Forward primer	Reverse primer
CRF	EF363131	CGA ACT CTT TCC CAT CAA CGT CCA	AGC GCC CTG ATG TTC CCA ACT TTA
CRFBP	GQ433718	ACT GAC CTC TGC ATC GCT TTC ACT	AAA CTT CCC ACT GGA CAC CAT CCT
CRF-R1	GQ433716	TTG GTG AAG GCT GTT ACC TCC ACA	ATG CCC TGA GTT TGG TCA TCA GGA
CRF-R2	GQ433717	TGC CAC AAC CGA TGA GAT TGG AAC	CGC TCC TCG TTG TGT TGT ACT TCA C
GnRH1	HBU31865	CAG ACA CAC TGG GCA ATA TG	GGC CAC ACT CGC AAG A
GnRH2	L27435	TGG ACT CCT TTG GCA CAT CAG AGA	CTC TGG CTA AGG CAT CCA GAA GAA
GnRH3	S63657	ATG GAT GGC TAC CAG GTG GAA AGA	TGG ATT TGG GCA TTT GCC TCA TCG
GnRH-R1	AY705931	TCA GTA CAG CGG CGA AAG	GCA TCT ACG GGC ATC ACG AT
GnRH-R2	AY028476	GGC TGC TCA GTT CCG AGT T	CGC ATC ACC ACC ATA CCA CT
AVT	AF517935	TTG GCT CCC TAG AAA CAG CTC ACT	TAC AGC CCT CAG AAT TGC AGC AGA
AVTR	AF517936	AGG AAC GAG GAG GTG GCA CAA ATA	AGG ACG CTT ACG TTC CCA ATC ACA
Somatostatin	AY585720	AGA AGA TCC TCC GAG CCG C	AGC TGA TGG AGG CGG TGA G
Actin	JF826504	CGC TCC TCG TGC TGT CTT C	TCT TCT CCA TGT CAT CCC AGT TG

### Analysis of RT-PCR data

Fluorescence readings for each sample were baseline subtracted and suitable fluorescence thresholds were automatically measured (MyiQ™ software). To determine the number of cycles needed to reach threshold, the original fluorescence reading data were analyzed using a curve-fitting RT- PCR algorithm [27]. This algorithm calculates reaction efficiency and the fractional cycle number at threshold of RT-PCR amplification curves providing a more accurate computation of initial cDNA concentration. All data are expressed as a ratio of gene of interest expression to actin expression.

### Statistical analysis

Comparison among behavior measures were conducted via two-way analysis of variance (two-way ANOVA; visual experience × behavior sampling time points) and followed by Tukey's post hoc analysis (SigmaStat 3.1, Systat Software Inc., San Jose, CA). Comparison among physiological samples, including circulating hormone concentration and gene expression levels was done using two-way ANOVA (visual experience × experimental groups), followed by Tukey's post hoc analysis. Within each experimental group (1, 3 and 7 day exposure), plasma hormone concentrations and gene expression levels of the experimental subjects were compared with controls by independent *t*-tests. For both experimental and control subjects, behavior, hormone levels, and gene expression levels were compared across different days by separate one-way ANOVA, followed by Tukey's post hoc analysis. Data are expressed as mean ± standard errors. The significant value was set as *p*<0.05.

## Results

### Visual cues suppress male dominant behavior for seven days

During the habituation period, both males established territories in their respective shelter half, and their coloration and behavior were those typical of dominant males. Prior to removal of the opaque barrier, experimental subjects and control animals showed no difference in DI (F_(1, 54)_ = 1.812, *p* = 0.184).

**Figure 2:**
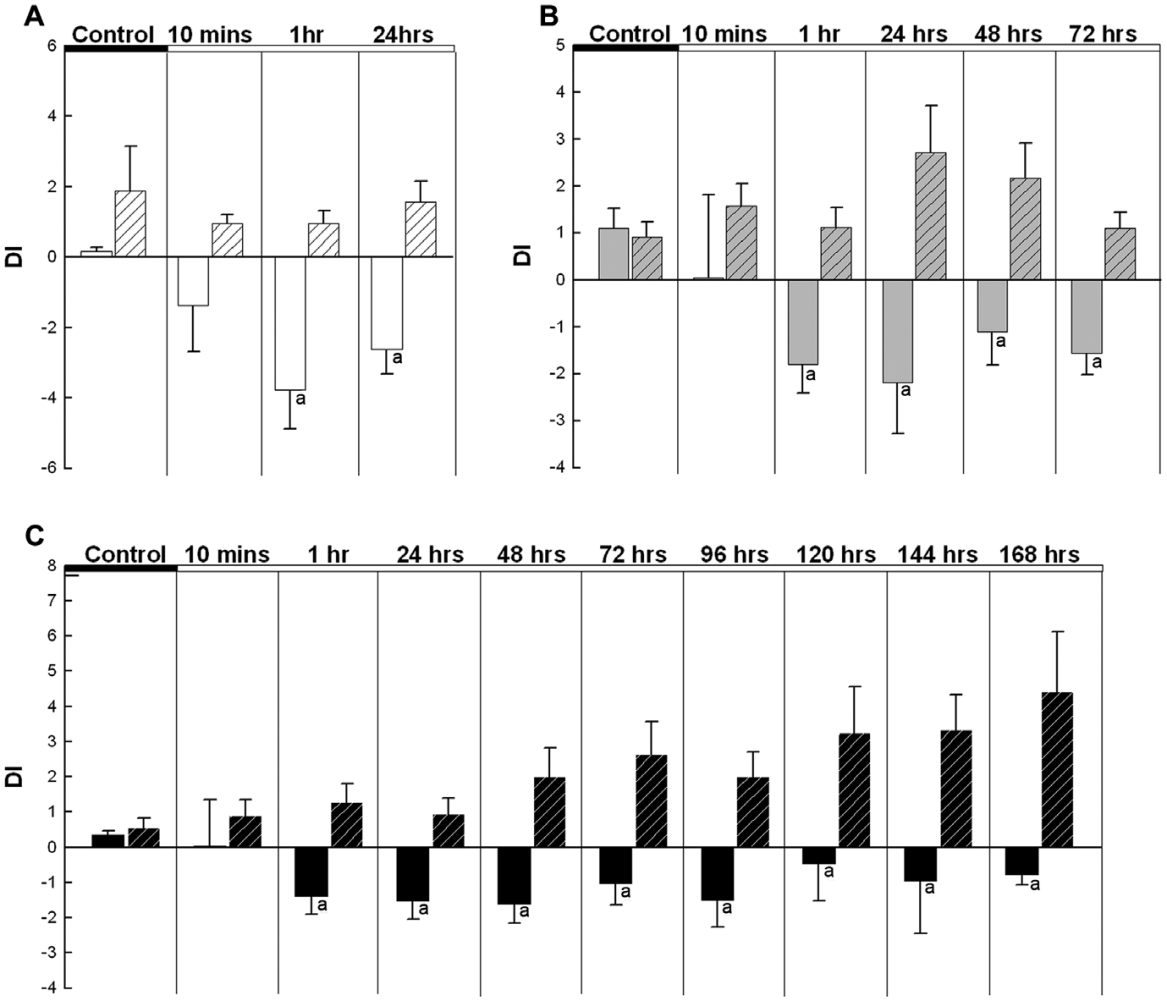
Bar graphs showing the mean dominance indices (DI) as a function of time. The results shown are: before visual exposure (control) and after visual exposure for three groups of animals up to 1 day (A), up to 3 days (B) and up to 7 days (C). Seeing aggressive acts by the larger conspecific male continuously suppressed the dominant behavior of the subjects. The subjects had decreased dominance indices one hour after seeing the aggressive stimuli in all three groups. The solid bars are subjects that were exposed visually to the larger stimulus male and the hatched bars are control subjects that saw no other fish. Mean values with letters are significantly different from corresponding mean values without letters. The standard errors (SE) of means are shown as error bars.

After removing the opaque barrier from the experimental tank, the two dominant males appeared to be sharing the same shelter as intended by the design of the pot arrangement (Figure 1), After the opaque barrier was removed, the stimulus male and subject male started fighting for territorial ownership by displaying dominant behaviors (including threat display, attack, border defense behaviors) through the clear barrier. When the subjects viewed an apparent attack from the larger dominant animal, they showed a consistent and significant decrease in DI (F_(9, 183)_ = 2.806, *p* = 0.004), in contrast to control animals (F_(9, 183)_ = 1.628, *p* = 0.11; Figure 2). Also, the colorful appearances of the subjects faded and the eye bar disappeared followed by the DI decrease in the experiments lasting 1, 3 and 7 days. This is the typical response of a male *A. burtoni* losing his territorial status. Thus, visual encounters alone suppressed the subject animal's dominant behaviors (two-way ANOVA main effect, F_(1, 380)_ = 61.524, *p*<0.001; Figure 2). This decrease in dominant behaviors was evident in the experiments lasting 1 day (F_(1, 72)_ = 29.935, *p*<0.001; Figure 2A), 3 days (F_(1, 108)_ = 29.935, *p* = 0.013; Figure 2B), and 7 days (F_(1, 180)_ = 34.721, *p*<0.001; Figure 2C). During the experiment, subjects increased the frequency of fleeing when they were “attacked” across the clear barrier (two-way ANOVA main effect, F_(1, 380)_ = 116.579, *p*<0.001), were drab colored without eye bar appearance, and tended to school with the female in their compartment, away from the shelter. Also, the stimulus male significantly increased his dominant behaviors immediately after removal of the opaque barrier (F_(9, 186)_ = 7.261, *p*<0.001) and maintained a similar level of dominant behavior during the entire experiment. These data show that a smaller subject acts like a subordinate male only in response to seeing the actions of the larger male. However, the behavioral responses of the subject were not correlated with the DI in any of the three experimental groups (*p* = 0.729; n = 198). This suggests that a visual stimulus of any intensity is sufficient to induce changes in behavior and physiology in the subjects that we describe below. Additionally those changes reflect a response pathway that is different from the full suite of changes that occur in response to uninhibited male-male encounters.

**Figure 3:**
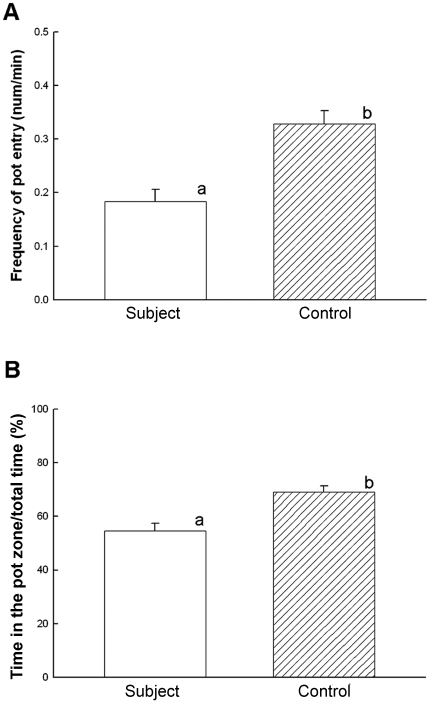
Seeing the larger conspecific male caused the subject to abandon his territory in the shelter. (**A**) The subjects reduced visits to the pot shelter (F_(1, 380)_ = 13.535, *p*<0.001) and (**B**) reduced the percentage of time spent in the pot zone out of total observation time (F_(1, 370)_ = 8.399, *p* = 0.004). Means with superscript letters are significantly different from those without letters. Error bars are the standard errors of means.

The subjects significantly reduced their entries to the shelter (two-way ANOVA main effect, F_(1, 380)_ = 13.535, *p*<0.001, Figure 3A) as well as a fraction of time near the shelter (F_(1, 370)_ = 8.399, *p* = 0.004, Figure 3B) after visually interacting with the larger male. However, the stimulus male spent a similar fraction of time spent near the shelter (around 90% time) during the entire experiment (F_(1, 179)_ = 1.532, *p* = 0.140) indicating that the larger male held his territory ownership. These data show that the larger stimulus male's visual presence alone resulted in the smaller male subject abandoning his territory and the half shelter despite absence of physical or chemical contact.

However, visual encounters alone did not significantly decrease gonadosomatic indexes [GSI  =  gonad size (g)/body weight (g)*100)], of subject males, although there was a trend in that direction (two-way ANOVA main effect, F_(1, 53)_ = 3.525, *p* = 0.066). This is in contrast to the reduction of GSI seen in subordinate animals in full contact with larger conspecifics [28]. Moreover, visual contact by large males did not significantly change the growth rates of experimental males in length (two-way ANOVA main effect, F_(1, 54)_ = 1.107, *p* = 0.297) or weight (two-way ANOVA main effect, F_(1, 54)_ = 0.116, *p* = 0.734) in contrast to measurement from animals in full contact [18]. Thus, visual stimuli alone from conspecifics suppress dominant behaviors and coloration, but not gonad size or growth rate.

### Visual information alone can change 11-KT levels during the first 24 hours

To examine the effect of visual interactions on reproduction and the stress responses during visual interactions, we measured the circulating levels of the stress hormone, cortisol, and male reproductive hormones including, testosterone (T), and 11 keto-testosterone (11-KT, a metabolic form of testosterone that is a functional androgen in teleost fish) of the subjects after 1, 3 and 7 days of exposure to visual threats. There were no significant differences in cortisol levels between the experimental and control subjects (two-way ANOVA main effect, F_(1, 49)_ = 0.0484, *p* = 0.827). However, cortisol level was negatively correlated with growth rate (Pearson correlation; coefficient correlation (r) = −0.289, *p* = 0.0326, n = 55), but not with DI (*p*>0.005; n = 55) in both experimental and control fish (Data not shown).

**Figure 4:**
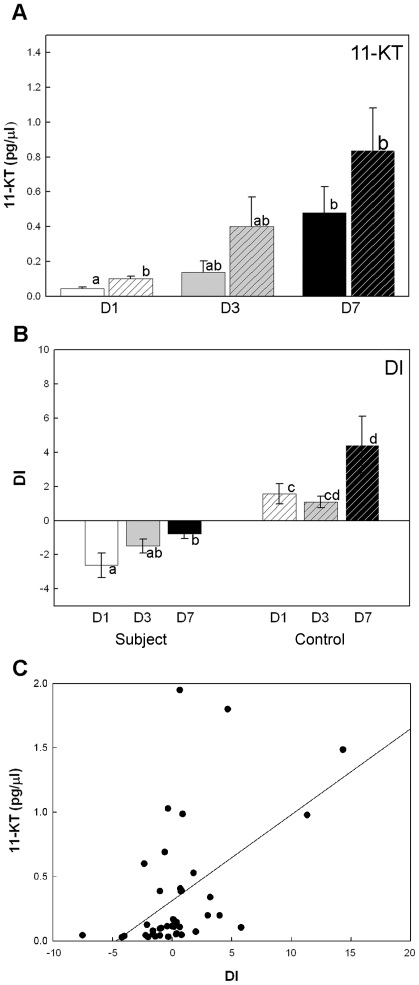
Circulating 11-KT concentrations were influenced by visual information and were correlated with dominant behaviors. (**A**) The circulating 11-KT concentrations were suppressed in the first 24 hours by the stimulus, and increased after 3 days in the new environment. The bars show the mean 11-KT (± SE) of the subjects (solid) and the controls (hatched) at day 1 (D1), day 3 (D3) and day 7 (D7). (**B**) The mean DI (± SE) as a function of groups. D1: Day 1 group; D3: Day3 group; D7: Day7 group. Means with no common superscript letters are significantly different. The standard errors of means are shown as error bars. (**C**) The DI was positively correlated with plasma 11-KT levels (r = 0.509, *p*<0.001, n = 58). The black dots represent the subjects, and the white dots represent the controls.

**Figure 5:**
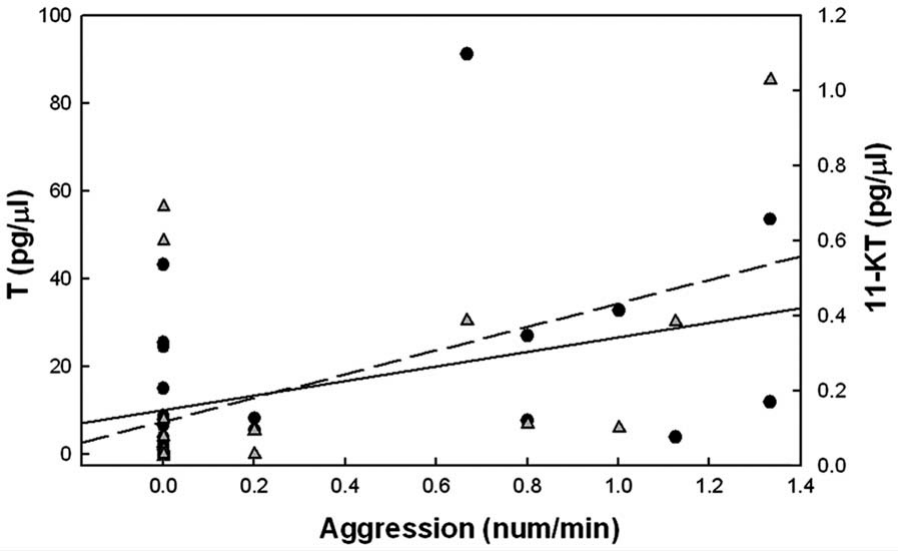
The frequency of aggressive behaviors was correlated with androgen concentrations in the plasma. The x-axis shows the frequency of all aggressive behaviors (chasing and border display). The T concentrations are shown on the left y-axis and were positively correlated with aggression (black circle; solid regression line; r = 0.384, *p* = 0.0438, n = 28). The 11-KT levels are shown on the right y-axis and were also positively correlated with aggression (gray triangles; dotted regression line; r = 0.506, *p* = 0.027, n = 19).

We found that T concentrations tended to be lower in the subjects who had encounters with larger conspecifics when compared to controls (two-way ANOVA main effect, F_(1, 49)_ = 3.191, *p* = 0.08). The T concentrations were positively correlated with reproductive behaviors in both experimental and control fish (r = 0.285, *p* = 0.0349, n = 55; Data not shown). The primary fish androgen, 11-KT differed significantly between the control and the experimental fish in the first 24 hours. Experimental subjects had lower levels of circulating 11-KT concentrations (t_13_ = −3.308, *p* = 0.005) than the control fish. The 11-KT levels of both control and experimental fish were higher in the 7-day experiment compared with the 1-day experiment (two-way ANOVA main effect, F_(2, 35)_ = 8.231, *p* = 0.001; Figure 4A). DI was lower in small experimental subjects after 7 days of exposure to larger neighbors (F_(2, 52)_ = 4.956, *p* = 0.011; Figure 4B). Furthermore, the circulating 11-KT concentrations in all subjects were correlated with DI (r = 0.509, *p*<0.001, n = 58; Figure 4C) and the frequency of aggressive behavior performances (r = 0.427, *p* = 0.005; Data not shown). In the experimental subjects, both 11-KT (r = 0.506, *p* = 0.027, n = 19) and T levels (r = 0.384, *p* = 0.0438, n = 28) were positively correlated with aggressive behaviors (Figure 5).

### Visual interactions change gene expression after three days of viewing a dominant male

 To understand the effects of visual encounters on gene expression in the brain-pituitary-gonad axis, we measured mRNA expression levels of several genes that are related to social status in *A. burtoni.*

**Figure 6:**
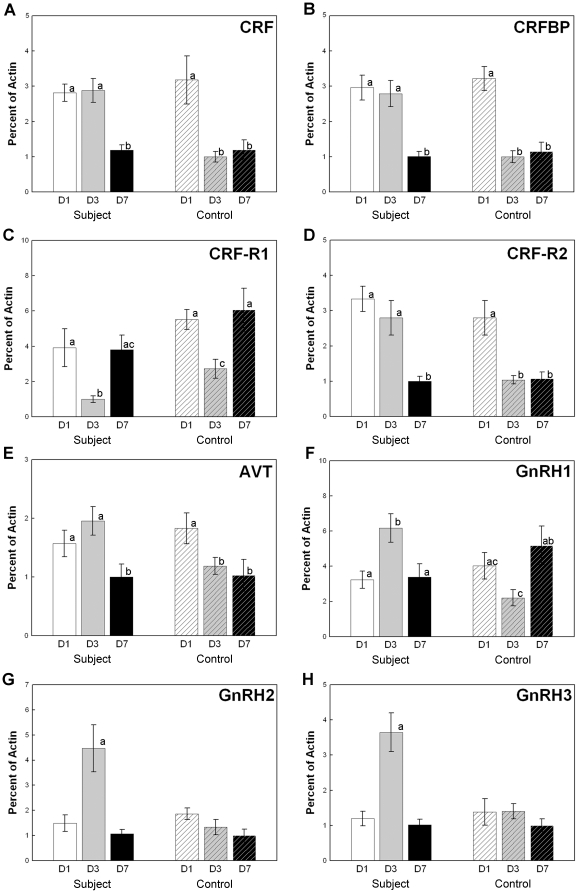
Brain gene expression levels were influenced by the visual stimulus after 3 days of exposure. Expression of stress related mRNAs, including CRF (A), CRFBP (B) and AVT (E) changed significantly. (C) CRF-R1 expression levels decreased following onset of visual threats, but (D) CRF-R2 expression levels increased. (F–H) Expression of the three GnRH mRNA levels increased following onset of visual threats at day 3. Means with superscript letters are significantly different from those without letters. Error bars show the standard error of the mean.

**Figure 7:**
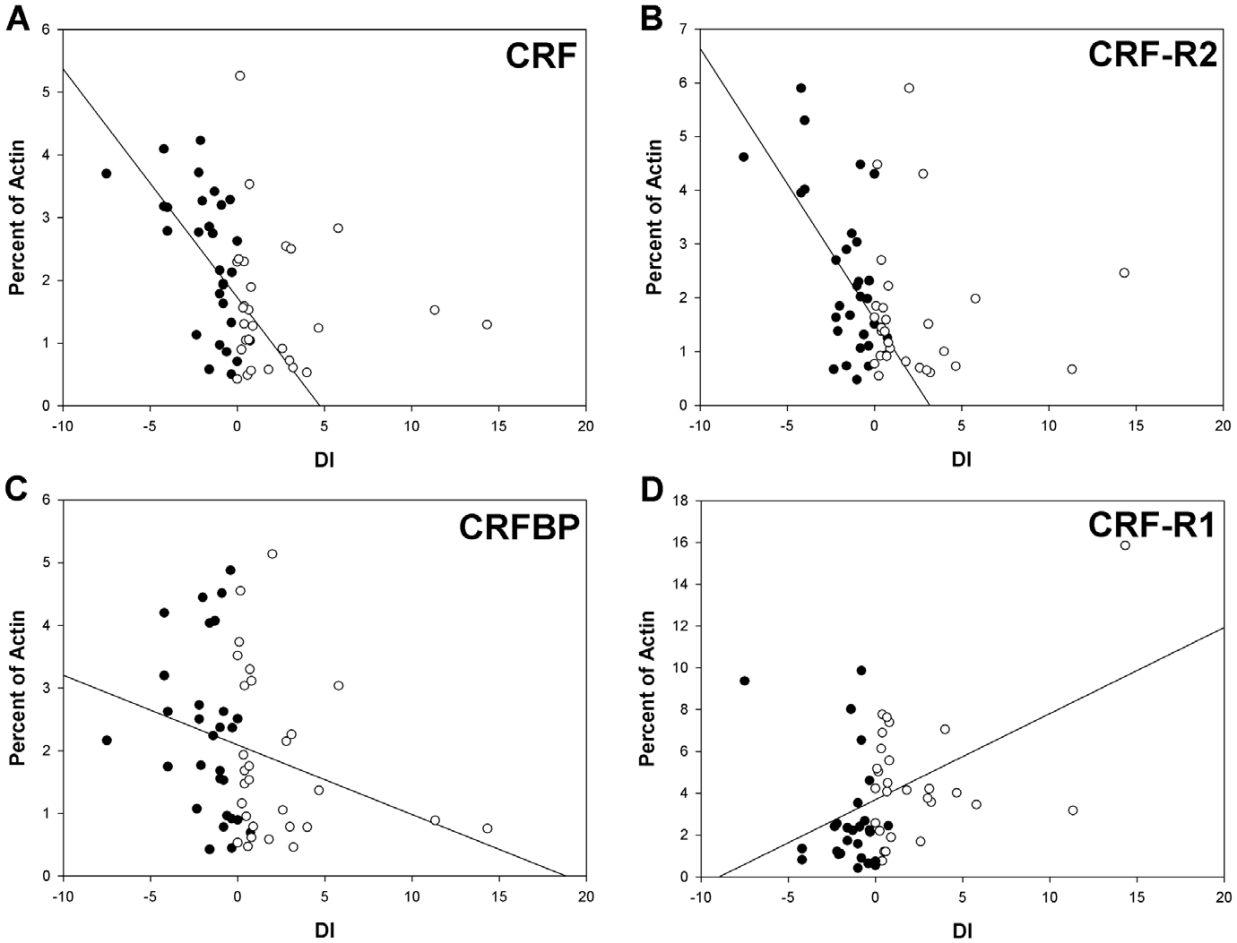
The CRF, CRFBP, CRF-R1 and CRF-R2 expression levels were significantly correlated with the dominance index (DI). (A) CRF and (B) CRF-R2 expression in the brains of experimental subjects was correlated with aggression (r = −0.562 and −0.584, *p*≤0.001, n = 29). (C) The total CRFBP expression levels were related to DI regardless of the visual experience (r = −0.278, *p* = 0.0347, n = 58). (D) In the control subjects, the CRF-R1 expression in the brain was related to dominance indices (r = 0.469, *p* = 0.0137, n = 27) and viewing the large conspecific male visually diminished this effect. The black dots represent the subjects, and the white dots represent the controls.

We measured gene expression levels of the CRF family in the brain and pituitary. Compared to the brain mRNA levels of the control group, the expression levels of the CRF family in the subjects were not different at 1 day and 7 days of exposure to a larger male. However, after three days of exposure to a larger male, mRNA levels were lower in the controls CRF (F_(2, 54)_ = 5.733, *p* = 0.006, n = 56; Figure 6A), CRFBP (F_(2, 54)_ = 8.062, *p*<0.001, n = 56; Figure 6B) and CRF-R2 (F_(2, 54)_ = 3.849, *p* = 0.027, n = 56; Figure 6D). This visual effect on CRF, CRFBP and CRF-R2 expression in the brain maintained the higher expression level was sustained until three days of exposure to a larger male. CRF-R1 mRNA level decreased in the subjects after three days of exposure to a larger male (Figure 6C). The mRNA levels of the CRF family in the brain were not related to cortisol levels in the circulation (Pearson correlation, *p*>0.05). These data suggest that the CRF gene family after 3-days of visual encounter could be related to some other functions, possibly the behavior changes during the visual encounter. CRF and CRF-R2 mRNA levels in the brain were correlated with escape behavior of experimental subjects from visual attacks by the larger male (Pearson correlation; coefficient correlation (r) = 0.583 and 0.551, *p*<0.001, n = 30) and negatively correlated with DI (r = −0.562 and −0.584, *p*<0.001, n = 29; Figure 7A and 7B). In addition, the CRFBP expression levels were negatively correlated with DI (r = −0.278, *p* = 0.0347, n = 28; Figure 7C). Conversely, the CRF-R1 expression in the brain was positively correlated with aggressive behavior (r = 0.404, *p* = 0.0322, n = 28) and DI (r = 0.469, *p* = 0.0137, n = 27; Figure 7D). These results indicate that during the visual encounter, the subject activates the CRF, CRF-R2 and CRFBP genes in the brain in response to fleeing from the social stressor. On the other hand, the decreased aggressive behavior is consistent with decreasing CRF-R1 expression in the brain.

We also examined the visual suppression of reproduction by measuring the gene expression level of the GnRH system in the brain and pituitary. The mRNA expression levels of all three fish GnRH ligands (GnRH1, GnRH2, GnRH3) in the experimental fish were significantly greater after three-days of visual exposure to large male fish compared with the control group (F_(2, 54)_ = 8.225, 8.89, and 9.206, *p*<0.001; Figure 6F–6H). In controls, the GnRH1 expression was lower on the third day and then recovered after one week (*p*<0.001). When an experimental subject was in visual contact with another much larger male, the GnRH1 expression was higher on the third day (*p* = 0.022) and then recovered after one week (*p*<0.001; Figure 6F). However, these changes were not correlated with the plasma concentration of T (r = −0.185, *p* = 0.177, n = 55) or 11-KT (r = 0.0446, *p* = 0.782, n = 41). Thus, the visual effect on GnRH activation appears not to be related to androgen production. In the pituitary, the gene expression of GnRH-R1 and GnRH-R2 were not significantly different between experimental and control subjects, but their levels were positively correlated with the androgens in the blood (T and GnRH-R1 in the pituitary, r = 0.366, *p* = 0.007, n = 53; 11-KT and GnRH-R1 or GnRH-R2 in the pituitary, r = 0.533 or 0.601, *p* = 0.004 or *p*<0.001, n = 40 or n = 40). These results suggest that the visual encounter couldn't fully suppress the reproductive axis.

To identify possible influences in gene expression related to behavioral changes, we also measured expression levels of mRNA from genes related to aggressive behaviors during social interaction, including arginine vasotocin (AVT), AVT receptor and somatostatin. AVT expression increased after visual exposure to a larger male (F_(2, 54)_ = 4.94, *p* = 0.011; Figure 6E). However, somatostatin and AVT receptor mRNA levels in the brain and pituitary were not affected by visual experience (*p*>0.05).

## Discussion

Social interactions can significantly influence behavior and physiology, typically via multiple sensory inputs. Here we tested the effects of visual exposure to a larger dominant male on a smaller, but also dominant male with a range of metrics from behavior to gene expression. We found that a smaller dominant male (subject) viewing a larger dominant animal (stimulus) changed both its behavior and physiology. Over a one-week observation period, social behavior and chromatic body markings were clearly influenced by visual stimuli. However, the visual components of social interactions did not mimic the full physiological effect that subordinates incur with physical contact. The experimental subjects reduced outward signals of any prior dominance but the typical concomitant physiological markers were not changed over the long term. This dissociation of key attributes of a socially dominant animal is striking. Are these animals minimizing the effects of visual threats by changing their appearance but maintaining their readiness to be dominant in the future?

In *A. burtoni*, non-territorial individuals typically exhibit subordinate behavior, including reduced aggression and locomotor activity as well as color changes [29], [30], [31], [32], [33], [34]. The color changes in our subject fish are consistent with loss of bright body coloration and eye bars in many cichlid and other fish species, which serves as a visual signal indicating social subordination [8], [35], [36], [37]. We found that the experimental subjects started the behavior and coloration changes consistent with subordinate status within 10 minutes to 1 hour after being visually exposed to larger males. This initial behavioral effect of subjects could be related to circulating androgen levels. In male teleosts, circulating androgen levels, especially 11-KT, are associated with reproductive and aggressive behaviors [38], [39], [40], [41], [42], [43], [44]. In *A. burtoni*, physical suppression by large dominant males and a loss of territorial status result in decreased circulating androgen levels in plasma [22], [45]. When animals were exposed to visual stimuli, we found that 11-KT concentrations were significantly lower than control groups on the first day, and correlated with the dominant behaviors. However, the visual effect on 11-KT concentrations disappeared after 3 days and the circulating 11-KT levels increased over seven days. This suggests that the 11-KT effect on aggressive behavior by visual contact alone weakened over time with a possible influence of the novel environment experienced after removal of the opaque barrier.

In *A. burtoni*, the subordinate males typically have reduced reproductive system capacity, including small gonad size and low levels of GnRH1 [46], [47], [48]. Following physical interactions between two territorial males in a prior experiment, GnRH1 expression levels of the loser and circulating androgen levels of the winner increased after 24 hours [49]. Here however, visual contact alone did not sustain suppression of GnRH1 expression in the brain and circulating androgen levels. The other two forms of GnRH ligands (type 2 and 3)[50] are not directly involved in androgen release but have been suggested to play a role in regulating reproductive behaviors, such as nest-building and spawning behavior [51]. Interestingly, the subject males in the present study had higher mRNA levels of all three types of GnRH but only on the third day after the onset of visual encounters. However, we did not find any correlation between GnRH ligands and reproductive behavior or androgen levels in our experiment. This suggests that these changes in gene expression by visual encounter did not lead to measureable changes in the brain-pituitary-gonad axis.

Subordinates typically activate CRF related genes in the HPI axis in response to social stress from dominant males. For example, stress induced CRF activation in the brain [52], [53], CRF receptor activation in the pituitary [32], [54], [55], and increased glucocorticoid hormones, in order to regulate subordinate behaviors [56], [57], [58]. In *A. burtoni*, circulating cortisol levels are higher in subordinate fish and quickly increase after physical encounters [23], [49]. Furthermore, during long-term social stress, subordinate males decrease CRF system activation in the brain and pituitary [26]. However, our study found that visual suppression is not sufficient to alter plasma cortisol levels or to decrease the CRF system activation in *A. burtoni* males.

Moreover, the changes in CRF family genes in the brain were correlated with aggression and escape behavior of experimental subjects, not circulating cortisol levels. This result indicates the visual information affects the CRF family in the brain for stress behavior regulation, as opposed to the endocrine functions in the HPI axis. Indeed, many studies have shown that all CRF family genes play a role in regulating behaviors under stress, including aggression, locomotion, and anxiety [24], [59], [60], [61], [62], [63]. However, the visual exposure to a social stressor could not maintain a long-term effect on the activation of the CRF family in coping with visual stress and modulating locomotion and anxiety. Changes in activation of the CRF family were present after 3 days of visual exposure, but disappeared after seven days of visual encounter. Perhaps if we looked at more discrete brain areas, we will be able to find a molecular difference during visual encounters.

Not all socially-regulated genes change their expression in the brain after three days of visual exposure (e.g., AVT and somatostatin). Many studies have shown that AVT is involved in social dominance and aggressive behaviors [42], [64], [65], [66], [67], [68]. Additionally, somatostatin is regulated by social status and induces aggression in male-male interactions in a cichlid [69]. However, all visual suppressed fish decrease aggressive behaviors to one week, but the changes of somatostatin and AVT levels do not seem to response that way they would if the subjects were getting physically attacked.

In sum, males visually exposed to a larger conspecific change their stress-coping strategy and apparently activate neural responses in response to the loss of status. Visual cues immediately change the androgen levels for regulating dominant behavior. Unlike the physical stress from conspecifics that can directly induce neural and hormonal changes within 24 hours [49], the visual stress weakened the neural responses against status loss on the third day. However, within one week, the visual suppression only existed in behavioral responses, rather than physiological changes, suggesting that visual encounters cannot completely alter social status that requires other sensory cues produced during physical contact. Visual information could play an important role in facilitating responses to social cues, but alone is not sufficient to physiological changes. Thus, these animals can uncouple the changes in circulating hormones from their effects on outward appearance. Perhaps the subject is presenting a false appearance outwardly that is not consonant with any internal changes.
